# Distribution, pathogenicity and disease control of *Fusarium tricinctum*

**DOI:** 10.3389/fmicb.2022.939927

**Published:** 2022-07-26

**Authors:** Yun Wang, Ruoyu Wang, Yuexia Sha

**Affiliations:** ^1^Key Laboratory of Desert and Desertification, Northwest Institute of Eco-Environment and Resources, Chinese Academy of Sciences, Lanzhou, China; ^2^Key Laboratory of Extreme Environmental Microbial Resources and Engineering, Lanzhou, China; ^3^Key Laboratory of Stress Physiology and Ecology in Cold and Arid Regions of Gansu Province, Lanzhou, China; ^4^Gaolan Station of Agricultural and Ecological Experiment, Northwest Institute of Eco-Environment and Resources, Chinese Academy of Sciences, Lanzhou, China; ^5^Institute of Plant Protection, Ningxia Academy of Agriculture and Forestry Sciences, Yinchuan, China

**Keywords:** distribution, pathogenicity, mycotoxins, disease control, *Fusarium tricinctum*

## Abstract

Plant pathogenic fungi such as *Fusarium tricinctum* cause various plant diseases worldwide, especially in temperate regions. In cereals, *F. tricinctum* is one of the most common species causing Fusarium head blight (FHB) and root rot. Infection with *F. tricinctum* results in high yield losses and reduction in quality, mainly due to mycotoxin contamination of grain. Mycotoxins produced by *F. tricinctum*, such as enniatins (ENs) and moniliformin (MON), which are the most studied mycotoxins, have been reported to have multiple toxic effects on humans and animals. Although chemical control of *Fusarium* infection has been applied to grains, it is not always effective in controlling disease or reducing the level of mycotoxins in wheat grains. To the contrary, chemical control may significantly increase infection of *F. tricinctum* in fungicide-treated plots after treatment. Our studies show that the bacterium *Bacillus amyloliquefaciens*, has good control effects against *F. tricinctum*. Therefore, its use as a biological control agent against various plant pathogens may be an effective strategy to control the spread of *Fusarium* pathogens. Here, we conduct a review of the literature involving this plant pathogen, its diversity, virulence, and methods to control.

## Introduction

*Fusarium tricinctum* is one of the most economically important plant pathogens and toxin-producing filamentous fungi in cereals and many other crops in the world (Marasas et al., 1967; Chelkowski et al., 1989; Andersen et al., 1996; Bottalico and Perrone, 2002; Kosiak et al., 2003; O’Donnell et al., 2013, Wiśniewska et al., 2014; Shi et al., 2017). Distribution of *Fusarium* species is dependent on climate and the genus is frequently observed within suitable climatic conditions. Temperature is the main climatic factor that affects the occurrence and development of *Fusarium* diseases of crops, although its impact of the climatic factor is not independent of other environmental and host factors (Saremi et al., 1999; Doohan et al., 2003). Among *Fusarium* species, *F. tricinctum* is thermal-specific pathogen, and lower temperature is beneficial to its activity and growth (Yan and Nelson, 2020). In fact, *F. tricinctum* is common in temperate regions, and usually appears as saprophyte or facultative parasite (Chelkowski et al., 1989; Andersen et al., 1996; Kosiak et al., 2003).

**FIGURE 1:**
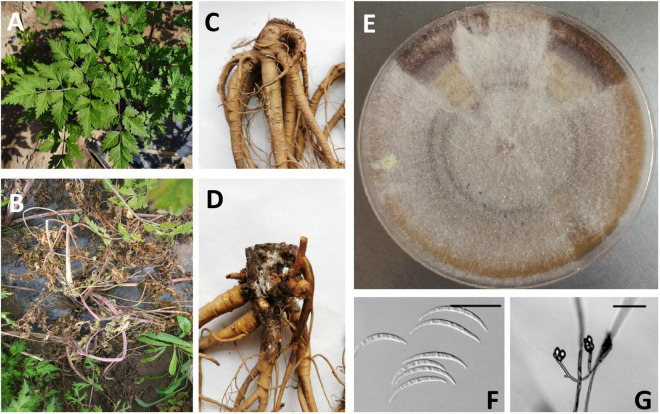
Observation of root rot symptom (*Angelica sinensis*) resulting from infection of *F. tricinctum*, above ground (**A:** healthy plant; **B:** infected plant) and below ground (**C:** healthy root; **D:** infected root); Colony of *F. tricinctum* on a PDA plate **(E)**; Micrograph image of *F. tricinctum* macroconidia (**F**, scale bar = 25 μm) and Microconidia (**G**, scale bar = 50 μm). **(A–E)** Photos taken by Dr. Liu of our group; **(F,G)** borrowed from Leslie and Summerell (2006).

*F. tricinctum* is related to *Fusarium* disease and prefer crop hosts, which is important to agriculture (Figures 1A–D). Studies have shown that *F. tricinctum* can potentially infect and colonize undamaged wheat leaves and produce conidia on senescent wheat leaves, resulting in wilt and “*Fusarium* head blight” (Wagacha et al., 2012), which can highly reduce crop yield. In addition, it is reported that *F. tricinctum* can produce mycotoxins as secondary metabolites, and the existence of mycotoxins in grains is a great worldwide concern. The presence of mycotoxins in feeds and foods is often associated with chronic or acute mycotoxin diseases in livestock and also in humans (Bottalico and Perrone, 2002).

Research on chemical control of *Fusarium* infection in grains has been reported. For example, use of tebuconazole fungicide has been effective against these fungal pathogens (Mesterhazy et al., 2003). However, fungicides containing tebuconazole and azoxystrobin are not always effective in controlling *Fusarium* disease, or reducing the levels of their mycotoxins in wheat grain (Pirgozliev et al., 2002; Wegulo et al., 2010). In addition, compared with untreated plots, *Fusarium* infection significantly increased in fungicide-treated plots, where the frequency of *F. tricinctum* was most often increased (Henriksen and Elen, 2005). These shortcomings require the search for alternative strategies, including biological control methods, to inhibit the prevalence of *Fusarium* pathogens. However, *Sphingomonas* and *Bacillus* bacteria may offer effective biological control of *Fusarium* pathogens (Dunlap et al., 2011; Wachowska et al., 2013; Shang et al., 2016).

*F. tricinctum* strains are saprophytes and plant pathogens of a variety of hosts including wheat and barley, and increasingly have been reported throughout the world (Torbati et al., 2019; Yan and Nelson, 2020; Senatore et al., 2021). However, the systematic synthesis of knowledge on these plant pathogens and more practical biocontrol methods are currently lacking. In this review, we will cover recent insights into the distribution of *F. tricinctum*, examine current understanding in pathogenicity, and discuss the strategies to control the disease caused by the pathogenic fungi.

## Taxonomy of *Fusarium tricinctum*

Corda first referred to *F. tricinctum* as *Selenosporium “tricnictum*,” and Saccardo transferred it to *F. tricinctum*, which are complex filamentous ascomycete fungi (Figures 1F,G), composed of many toxin-producing plant pathogens with important agricultural significance, then it was neotypified by Neish (1987). Holubová-Jechová et al. (1994) assigned epitypification for this species. *F. tricinctum* grow rapidly on PDA, forming a large number of dense mycelia that are initially white, but will become pink, red or purple with age, since they can form red pigments in agar (Figure 1E). *F. tricinctum* can be distinguished from some closely related species in terms of the macroconidial shape and the monophialidic conidiogenous cell. Cultures of *F. tricinctum* can be confused easily with *F. graminearum*, *F. pseudograminearum*, and *F. culmorum* (Leslie and Summerell, 2006). Differences in the morphology of the macroconidia allow the differentiation of *F. tricinctum* from *F. graminearum*, *F. pseudograminearum*, and *F. culmorum*. More importantly, as a member of the Section *Sporotrichiella*, the presence of microconidia distinguishes isolates of *F. tricinctum* from isolates of which form colonies on PDA similar to those of *F. tricinctum*. In addition, *F. tricinctum* cannot produce polyphialides, which is different from some close relatives (Leslie and Summerell, 2006).

**FIGURE 2:**
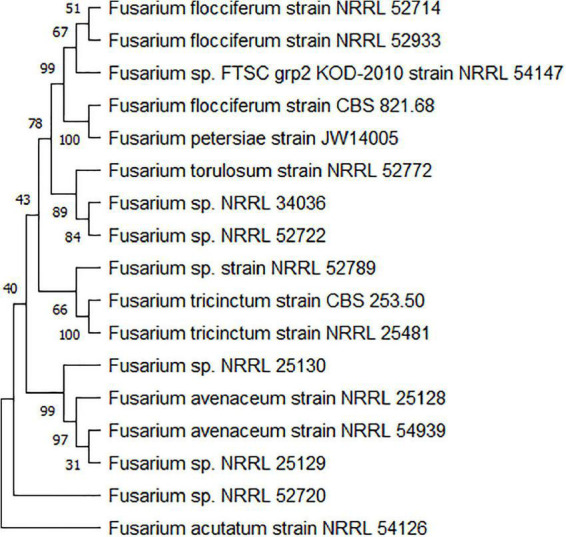
Maximum-likelihood (ML) phylograms obtained from the combined partial *TEF* and *RPB2* data set (2,487 bp) of the *Fusarium tricinctum* species complex (FTSC) isolates. Sequences were used to conduct BLASTn queries of NCBI GenBank (https://www.ncbi.nlm.nih.gov/). Aligned sequences of 17 FTSC reference strains were combined and analyzed via ML bootstrapping using MEGA 11. Bootstrap values (%) are shown on clades.

*F. tricinctum* has a very close relationship with *F. avenaceum*, which together with other *Fusarium tricinctum* species complex (FTSC), are related with Fusarium head blight (FHB) and seedling diseases (stem and root rot) of all cereals (Bottalico and Perrone, 2002). FTSC members include isolates of *F. avenaceum*, *F. flocciferum*, *F. petersiae*, *F. acuminatum*, *F. tricinctum*, and other unclassified FTSC (NCBI Taxonomy, Figure 2). New members belonging to FTSC were described as *F. gamsii* and *F. iranicum* from Iran (Torbati et al., 2019), and as FTSC 12, 13, 14, 15 from Italy (Senatore et al., 2021). Members of the FTSC complex produce a number of “emerging” mycotoxins, including enniatins (ENs) and moniformin (MON) that may pose a threat to food safety and human health (Jestoi, 2008). Phylogenetic analysis has shown that the genetic relationship among these species is quite close (Turner et al., 1998), and it is very difficult to distinguish them from each other on the basis of morphological and physiological characteristics, but they can be distinguished by means of molecular methods. DNA sequence data from several marker loci have been used to resolve phylogenetic relationship within the FTSC (Figure 2), including *ACL1* (ATP citrate lyase 1), *TUB-2* (β-tubulin), *ITS* rDNA, *ESYN1* (enniatin synthetase 1), *RPB1* (RNA polymerase subunit 1, *RPB2* (RNA polymerase subunit 2), and *TEF1* (translation elongation factor 1α) (Turner et al., 1998; Kristensen et al., 2005; Kulik et al., 2007; Niessen et al., 2012; Senatore et al., 2021).

## The distribution and ecology of *Fusarium tricinctum*

**FIGURE 3:**
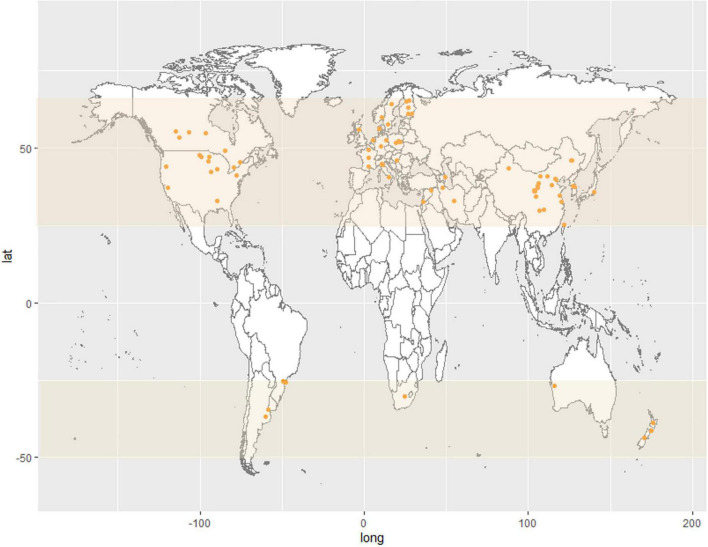
Map showing the global distribution (orange dots) of plant hosts with reported *F. tricinctum* infection. The distribution is limited to the temperate zone (covered in light yellow).

*F. tricinctum* is plant pathogen all over the world that have the potential to infect and colonize various cereal crops, such as wheat, rice, maize, and oats in temperate and also semi-tropical cereal-growing areas, including Asia, North America, South Africa, and all Europe (Marasas et al., 1967; Lamprecht et al., 1988; Chelkowski et al., 1989; Andersen et al., 1996; Golinski et al., 1996; Kosiak et al., 2003; Wiśniewska et al., 2014; Shi et al., 2017). *F. tricinctum* was recently reported as plant pathogen in Argentina, Brazil, Western Australia, and South Australia (Castañares et al., 2010; Barkat et al., 2016; Moreira et al., 2019; Supplementary Table 1 and Figure 3).

*F. tricinctum* was isolated from the diseased stem bases of wheat and was the most frequent species of *Fusarium* isolated from malted barley in Denmark (Andersen et al., 1996) and from winter wheat in Sweden (Lindblad et al., 2013). Also, it rarely occurred in pods and seeds of mature plants or residue (Nyvall, 1976). *F. tricinctum* is usually isolated from moldy corn, fescue, and most small grains (Marasas et al., 1967; Bamburg et al., 1968; Burmeister and Hesseltine, 1970). It is also the cause of postharvest rot of onion bulbs and pumpkins (Carrieri et al., 2013), as well as dry rot of seed tubers of potatoes in Michigan (Aktaruzzaman et al., 2018). In addition, *F. tricinctum* was found as endophytic species in healthy trees and healthy seedlings of Persian oak trees in Iran (Alidadi et al., 2019; Supplementary Table 1). Our work proved that *F. tricinctum* was the pathogen causing bulbs rotting and spalling from the basal disc, as well as progressive yellowing and defoliation of the leaves in lilies and Lanzhou lilies (Li et al., 2013; Shang et al., 2014). Interestingly, we also found that *F. tricinctum* was involved in root rot of Chinese herbal medicine, e.g., *Angelica sinensis* and wolfberry (Liu et al., 2021; Uwaremwe et al., 2022).

Geographic distribution of *F. tricinctum* appears to be related to climatic conditions, such as temperature and humidity. Climate is the main factor that influences the distribution of *Fusarium* in soil, and the effects of temperature on the colonization of roots and soil propagule density have also been experimentally determined to be a factor (Saremi et al., 1999). The *Fusarium* species differ in their climatic distribution and optimum climatic conditions are required for their persistence. Temperature and humidity are the main climatic factors that affect the occurrence of grain *Fusarium* diseases, although the influence of these climatic factors is not independent of other environmental and host factors. Conditions that are conductive to *in vitro* growth are usually the most favorable for production of mycotoxins in cereal crops (Doohan et al., 2003).

The incidence of the pathogenic organisms of wheat *Fusarium*, barley *Fusarium*, and maize ear rot are often related to different climatic conditions (temperature and rainfall) in different geographic locations. Temperature has a significant influence on *Fusarium* root rot of soybeans, and *F. tricinctum* is the major pathogen of the root rot disease and show obvious thermal-specific (Yan and Nelson, 2020). The cooler temperatures might be conductive to the activity of *F. tricinctum* (Yan and Nelson, 2020). *Fusarium* species are geographically distributed soil fungi because weather has an important influence on the abundance and activity of the species (Saremi et al., 1999; Doohan et al., 2003). *F. tricinctum* usually also appears as a saprophyte or a parasite of plants in temperate and semi-tropical regions (Figure 3; Chelkowski et al., 1989; Andersen et al., 1996; Kosiak et al., 2003).

Even before infecting the host, climate has the potential to affect the incidence and severity of *Fusarium* disease. Once the *F. tricinctum* inoculum disperses to the host, climate factors, temperature, and humidity play a vital role in the infection and colonization of grains by *F. tricinctum* (Doohan et al., 2003). Temperature or osmotic stress may indirectly affect the development of disease by inducing the host antifungal defensive mechanisms before attacking of pathogen (Conrath et al., 2002). Although *in vitro* growth tests showed that *Fusarium* have temperature ecotypes on the basis of species and origin location, *in vitro* pathogenicity tests suggested that species is much more important than climatic origin in determining the pathogenicity of *Fusarium*, regardless of the temperature (Brennan et al., 2003).

## The pathogenicity from *Fusarium tricinctum*

*F. tricinctum* is related to *Fusarium* disease, which can reduce crop yield and cause the accumulation of mycotoxins in grain products. The potential of *Fusarium* to infect and colonize undamaged wheat leaves, and the potential to produce conidia on senescent leaves cultured were investigated. Studies have also shown that *F. tricinctum* forms sporophores and erupts through the leaf surface to release a large number conidia, resulting in wilt and FHB of cereal crops, such as wheat, oat, barley, and maize (Wagacha et al., 2012).

Many of *Fusarium* species are well-known for their mycotoxins produced as secondary metabolites, and the presence of mycotoxins in feeds and foods is often associated with chronic or acute mycotoxin diseases in livestock and also in humans. However, there is a great variability in the production of biologically active secondary metabolites between species (Visconti et al., 1992). The main groups mycotoxins commonly found in *F. tricinctum* are: ENs, MON, and T-2 toxin, which are currently covered by limited literature (Langseth, 1998; D’mello et al., 1999; Thrane, 2001; Jestoi, 2008). ENs, the most studied mycotoxins, produced by *F. tricinctum* are usually considered to be less toxic than trichothecenes and are known as a phytopathogenic compound causing necrosis and wilt (Cuomo et al., 2013). MON has been frequently purified from cultures of *F. tricinctum*, and the role of MON (a potassium or sodium salt of a cyclobutene) is to inhibit enzymatic systems and gluconeogenesis (Jestoi et al., 2004). The most reported trichothecenes mycotoxin in *F. tricinctum* is T-2 toxin, which is associated with acute toxicity (Prelusky et al., 1994). In addition, some of the mycotoxins may also have the potential to be applied as drugs or drug candidates. These mycotoxins include fusarielins (Hemphill et al., 2017), Visoltricin (Visconti et al., 1992), and ENs (Wätjen et al., 2009), which are medicinally used as antibiotics for the treatment of nasopharyngitis (Hemphill et al., 2017).

**FIGURE 4:**
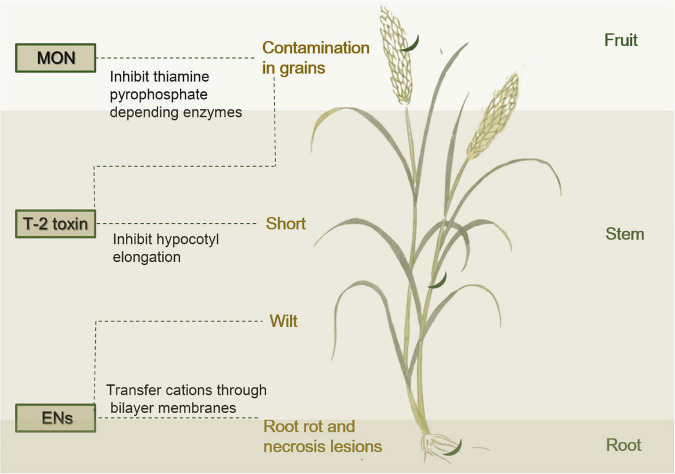
Diagram that *F. tricinctum* (crescent moon in dark green) infects a plant, releases mycotoxins and causes disease symptoms in plants.

**Enniatins** (ENs) are a group of fungal mycotoxins with a hexadepsipeptidic chemical structure and they possess many potent biological activities that can contaminate a variety of foodstuffs increasing the exposure risk for consumers (Cuomo et al., 2013). Among FHB pathogens of cereals, *F. tricinctum* are the most effective ENs producers in naturally contaminated grain (Jestoi et al., 2004; Jestoi, 2008). The cyclic hexadepsipeptide compounds are known as phytopathogenic toxins from *F. tricinctum* causing symptoms such as necrosis lesions, rot, and wilt (Hornbogen et al., 2002; Figure 4). The molecule consists of three alternating residues each of a branched chain amino acid and D-hydroxyisovaleric acid (Supplementary Figure 1). ENs are synthesized by a 347 kDa multienzyme (EN synthetase), and the corresponding gene *esynl* has an open reading frame of 9,393 nucleotides (Hornbogen et al., 2002). The biological activities of the ENs are largely due to their ability to transfer cations through bilayer membranes without forming membrane pores (Jestoi, 2008). ENs are then integrated into cell membranes, forming passive cation-selective channels (Cuomo et al., 2013). ENs contribute to the wilt toxic character of *F. tricinctum*, and their virulence was significantly reduced after disruption of the *esynl* gene (Hornbogen et al., 2002). In addition to phytotoxicity, ENs show antimicrobial, insecticidal, herbicidal, and anthelminthic activities (Burmeister and Plattner, 1987; Herrmann et al., 1996; Jeschke et al., 2003; Uhlig et al., 2007), as well as high cytotoxicity to mammalian cells (Jestoi, 2008). ENs A1 and B1 and, to a lesser extent, enniatin B may possess anticarcinogenic properties by induction of apoptosis and disruption of extracellular regulated protein kinase signaling pathway. Further analysis of ENs is necessary to investigate their potential importance for cancer therapy (Wätjen et al., 2009).

**Beauvericin (BEA)** is a cyclodepsipeptide metabolite, closely related and co-occurred with ENs (Hornbogen et al., 2002; Supplementary Figure 1). BEA was currently reported to be associated with the presence of *F. tricinctum* (Hellin et al., 2016), and is a very potent channel-forming molecule as it induces pores in lipid membranes (Moretti et al., 2002). The non-selective toxic activity of BEA could be explained by its capacity to induce biological membrane pores, resulting in interference with the normal gradients of physiologically important monovalent cations on the cell membranes (Moretti et al., 2002). This process could determine the role of BEA in pathogenicity caused by *F. tricinctum* strains that produce the toxin. However, *F. tricinctum* strains do not produce very high levels of BEA (Uhlig et al., 2007). BEA is not only highly toxic to insects (Gupta et al., 1991), but also cytotoxic to mammalian cells and tissues, and has been reported to cause apoptosis in mouse and human cell lines (Macchia et al., 1995).

**Moniliformin** (MON) was structurally characterized and identified as the sodium salt of the semisquaric acid, and the relatively small semisquaric anion can be assumed to chemically behave similarly to inorganic anions (Supplementary Figure 2; Springer et al., 1974). Severe infections of *F. tricinctum*, were usually responsible for MON occurring in scabby grains, according to the surveys from some European countries (Kostecki et al., 1995). In Austria, MON occurred in freshly harvested durum wheat (Adler et al., 1995). The contamination MON in grains was closely associated with the presence of *F. tricinctum* in most surveys (Kostecki et al., 1995; Bottalico and Perrone, 2002; Figure 4). MON acts as an inhibitor of thiamine pyrophosphate depending enzymes, such as pyruvate dehydrogenase, ketoglutarate dehydrogenase, and pyruvate decarboxylase (Gathercole et al., 1986; Pirrung et al., 1996). In addition, MON was reported to inhibit glutathione peroxidase, aldose reductase, reductase, and gluconeogenesis (Deruiter et al., 1993; Wu and Vesonder, 1997). MON in diets was associated with reduced performance, hematological diseases, myocardial hypertrophy, and mortality in pigs and rodents (Harvey et al., 1997; Bottalico and Perrone, 2002), as well as muscular weakness and acidosis in poultry (Nagaraj et al., 1996). The major target organs of the mycotoxin are the cardiac and skeletal muscles (Harvey et al., 2002). Long-term exposure to MON has been associated with reduced weight gain, heart muscle damage, and disordered immune system in some laboratory animals (Harvey et al., 1997, 2002; Li et al., 2000). MON has been speculated to be associated with outbreaks of human Keshan disease in some areas of China where ingestion of MON-contaminated maize has caused cardiac lesions (Yu et al., 1995; Uhlig et al., 2007).

**T-2 toxin** belongs to type A trichothecenes, and is one of the most toxic mycotoxins (Prelusky et al., 1994). T-2 toxin is usually produced at low temperatures by the *F. tricinctum* (Burmeister, 1971). T-2 toxin can inhibit hypocotyl elongation of soybean (Figure 4). Although the inhibition of elongation by cytokinin was similar to that of T-2 toxin, the two compounds appeared to act in different ways (Stahl et al., 1973). Rats fed the T-2 toxin diet were severely stunted with inflammations of the skin around the mouth and nose (Burmeister, 1971), while a large animal (steer) that received daily intramuscular injections of T-2 toxin lost weight during the study and died after long-term treatment (Grove et al., 1970).

**Fusarielins** have received little attention in the *Fusarium* community as metabolite group. The ability of the *Fusarium* species to produce fusarielins is largely unknown. Fusarielins A and B were isolated from a *F. tricinctum* strain (Nenkep et al., 2010). Fusarielins have been shown to interfere with the microtubule function (Kobayashi et al., 1995). In antibacterial assays, it was found that fusarielins A and B were both mild antibiotics (Nguyen et al., 2007). Fusarielins have also been shown to have toxic effect on human epithelial carcinoma cell lines, and to be characterized as mycoestrogens, as they stimulate growth of MCF-7 breast cancer cells (Kobayashi et al., 1995; Sørensen et al., 2012).

On the other hand, the pathogenicity of *F. tricinctum* may be used to control the spread of invasive weeds. The annual herb *Bromus tectorum* (cheatgrass) has been becoming a serious invasive plant in semi-arid habitats of Northwestern America in winter. *F. tricinctum* isolated from the local soils is the pathogen of *B. tectorum* seeds, and is the main reason for the complete death of the invasive grass (Masi et al., 2017). *F. tricinctum* produce a large number of phytotoxins that participate in the pathogenesis (O’Donnell et al., 2013). Acuminatopyrone and blumenol A showed a significant inhibitory effect on the radicle length of cheatgrass seedlings (Masi et al., 2017). *F. tricinctum* that can cause *B. tectorum* infection and death show potential as biocontrol agents against invasive weeds (Masi et al., 2017).

## Disease control to *Fusarium tricinctum*

Some studies have been reported on the chemical control of *Fusarium* infection in grains (Henriksen and Elen, 2005). Treatment of *F. tricinctum* cultures with dilute sodium bicarbonate can significantly reduce the production of trichothecene mycotoxins, geraniol, and carotenoids (Roinestad et al., 1994). Chlorine dioxide (ClO_2_) is a powerful disinfectant with a wide range of high biocidal activity. The treatment with aqueous ClO_2_ significantly reduced the populations of *F. tricinctum* and prevented the occurrence of chestnut kernel rot (Chen and Zhu, 2011). In addition, some natural plant extracts have the effect of inhibiting the growth of *F. tricinctum* under laboratory conditions. *Curcuma longa* extract is related to the destruction of the synthesis of key proteins and enzymes of fungal cell membrane systems, which may inhibit the synthesis of ergosterol and the respiratory chain, ultimately inhibiting the growth of *F. tricinctum*. Certain chemical components of *C*. *longa* have the potential to be developed as a series of environmentally sustainable bio-fungicides (Chen et al., 2018). The best results were obtained after artificial inoculation tests with fungicide tebuconazolein (Mesterhazy et al., 2003). However, compared with untreated plots, a significant increase of *Fusarium* infectious was detected in fungicide treated plots. *F. tricinctum* was the most frequent species detected after fungicide treating (Henriksen and Elen, 2005). The effect of fungicides on *Fusarium* grain infection was studied in the Norway field trial. Significant increase in *Fusarium* infection was detected in fungicide-treated plots compared with untreated plots. The level of the dominating species (*F. tricinctum*) increased after fungicide application. Other *Fusarium* species were detected only in low frequencies. In the fungicide trials, Diamant (epoxyconazole + kresoximmetyl) and F9215 (spiroketalamin + tebuconazole) increased *F. tricinctum* significantly, while there were no significant effects on the other *Fusarium* species (Henriksen and Elen, 2005). According to some reports, fungicides containing tebuconazole and azoxystrobin were not always effective in controlling *Fusarium* disease in wheat or reducing mycotoxin levels in grain products (Pirgozliev et al., 2002; Wegulo et al., 2010). These shortcomings require the search for alternative strategies, including biological methods, to control the spread of *Fusarium* pathogens.

Diverse microorganisms may contribute to the biological control of plant pathogenic microbes, and most research work has focused on isolates of some bacteria genera (Mcspadden Gardener and Driks, 2004). The controlling effects of *Sphingomonas* and *Bacillus* on winter wheat colonization pathogens, were studied under laboratory conditions. The *Sphingomonas S 11* isolate has an antagonistic effect on *F. tricinctum*. The infection symptoms of winter wheat seedlings treated with a suspension of *Sphingomonas S 11* bacteria and inoculated with *Fusarium* pathogens were significantly lower than those of unprotected seedlings that were inoculated with the above mentioned pathogens (Wachowska et al., 2013).

*Bacillus* can produce a broad spectrum of antimicrobial compounds, and this activity makes them candidates as biological control agents against a variety of plant pathogens (Mannanov and Sattarova, 2001; Mcspadden Gardener and Driks, 2004). The biological activity of these strains is often related to the production of secondary metabolites, such as antimicrobial cyclic lipopeptides (Dunlap et al., 2011). Our research shows that *Bacillus amyloliquefaciens* has good control effects on root rot infested by *F. tricinctum* in Chinese herbal medicine of *Angelica sinensis*, Lanzhou Lilies, and wolfberries (Shang et al., 2016; Liu et al., 2021; Uwaremwe et al., 2022). *B. amyloliquefaciens* strains were found to inhibit *F. tricinctum* fungal mycelial growth, *in vitro* and *in planta*, as well as to promote the growth of seedlings (Liu et al., 2021; Uwaremwe et al., 2022).

Whether organic farming can control the activity of *F. tricinctum* has not been reported. Bernhoft et al. (2010) found that the infection rate of *F. tricinctum* in organically produced wheat was lower, and the infestation and mycotoxin levels of *Fusarium* found in organic grains was lower as well (Bernhoft et al., 2010). However, it remains unclear whether organic amendments technology is effective in disease control and suppression, and farmers have often neglected its role in disease management. The control of onion pink rot by organic amendments is not easy to predict and apply on a large scale (Carrieri et al., 2013). In addition, organic solarization amendments cannot completely eliminate the *Fusarium* population in the soil, as evidenced by the existence of several infectious onion bulbs in solarized treatments (Carrieri et al., 2013). The agricultural intensity index includes the application amount rate of pesticide and nitrogen fertilizer, which may reflect some important differences between organic and conventional agricultural systems. It has been reported that agricultural intensity has an obvious impact on the community structure of *Fusarium* in wheat grains. Importantly, agricultural intensity increased the abundance of *F. tricinctum* (Karlsson et al., 2017).

The agroforestry system is a multi-functional plant production system and has attracted attention as a sustainable method that can replace traditional monoculture agriculture. The colonization rate of wheat grain in agroforestry combined with the FHB pathogens *F. tricinctum* was lower than that in conventional monoculture. Therefore, the biological control of *F. tricinctum* in wheat grain may be enhanced due to diversification under agroforestry practices (Beule et al., 2019). In addition, altering the microclimate conditions may inhibit the infection of *F. tricinctum* in the diversification of agroforestry systems.

## Interactions with other *Fusarium* species

A full-scale understanding of the interactions between *Fusarium* species in grains gives us a better view of the ecological role of *F. tricinctum*, which is greatly important for limiting *Fusarium* disease and mycotoxin contamination in crops. There may be both synergistic and competitive interactions within *Fusarium* communities, *F. poae* and *F. tricinctum*, the pair of *Fusarium* species co-existing in Swedish farmland (Karlsson et al., 2017). *F. tricinctum* and *F. langsethiae* were highly correlated in mature cereals in Belgium (Hellin et al., 2016). Two *Fusarium* species prefer to share the same environmental conditions instead of direct inter-species interactions, that is, symbiosis mode may be reliant upon the external associations. In addition, the co-inoculation of multiple *Fusarium* species led to competition in a controlled experiment, and the competitive interaction resulted in a decrease in fungal biomass and an increase in the amount of mycotoxin (Xu et al., 2007).

Management of *Fusarium* disease is complicated, due to the complexity of *Fusarium* species involved in an infection. Paired *Fusarium* cultures have different interactions between different isolates (Wagacha et al., 2012). The interaction between species may partly rely on the type of mycotoxins produced during the infection process (Llorens et al., 2006). Compared with deoxynivalenol (DON), ENs is much less phytotoxic to wheat and may has different effects on competing microorganisms. The individual mycotoxins may cause relative competition between species under certain circumstances. However, it is worth noting that other factors, including host plant species, climate factors, and other environmental conditions, may also play an important role in the interaction between *Fusarium* species (Llorens et al., 2006; Wagacha et al., 2012).

## Future prospects

*Fusarium* disease is destructive for crops. The quality of grains deteriorated due to contamination by a series of mycotoxins produced by *Fusarium*. Although the disease has economic significance, disease control and prediction are still difficult due to the variety of *Fusarium* species involved. Different species may have different responses to different control measures, and also may have different interactions (competitive or synergistic) between species. Therefore, a comprehensive understanding of the ecological role of *F. tricinctum* at the community level is important in agricultural practices.

Such exploratory work requires relatively large amount of investment and may lead to the improvement of powerful new facilities for the research and application of *Bacillus*-mediated biological control. Obviously, this is an exciting time for basic research on plant–microbe interactions and microbiological ecology, as well as for efforts to improve agricultural technologies. We hope that this information will stimulate new research and will eventually lead to the wider application of safe and effective biocontrol agents, thereby promoting plant health.

## Author contributions

YW: literature collection, processing, interpretation, writing, and submitting. YS: sequences blasting and phylogenetic analysis. RW: project coordination and supervising. All authors contributed to the article and approved the submitted version.

## Conflict of interest

The authors declare that the research was conducted in the absence of any commercial or financial relationships that could be construed as a potential conflict of interest.

## Publisher’s note

All claims expressed in this article are solely those of the authors and do not necessarily represent those of their affiliated organizations, or those of the publisher, the editors and the reviewers. Any product that may be evaluated in this article, or claim that may be made by its manufacturer, is not guaranteed or endorsed by the publisher.
