# Supplementary material for: Distribution, pathogenicity and disease control of Fusarium tricinctum
Source: Front Microbiol. 2022 Jul 26;13:939927. doi: 10.3389/fmicb.2022.939927 (PMC9360978; doi:10.3389/fmicb.2022.939927)
Supplement: Supplementary file 1 [file Data_Sheet_1.PDF]

Table S1. The main reported geographical origin and hosts of *Fusarium tricinctum*

Geographical origin	*Symptoms of infection*	*Host or source*	*References*
Poland	Severely pathogenic towards cereal seedlings	Cereals; wheat heads	(Chelkowski et al., 1989b); (Wiśniewska et al., 2014)
Brazil	Fusarium Head Blight	Wheat	(Moreira et al., 2019)
Norway	Potential risk of mycotoxin contamination in grains	Barley, oats and wheat	(Kosiak et al., 2003b)
Sweden	Mycotoxin contamination in seeds	Oats	(Fredlund et al., 2013a)
North Dakota, US	Reduced emergence of seedlings; reduced plant height, number of plants with trifoliate leaves, and root biomass; yield reduction	Soybean	(Chitrampalam & Nelson, 2014)
Ningxia, China*	Wilting, brown spots of bulbs , bulbs rotting and spalling from the basal disc, plus a progressive yellowing and defoliation of the leaves	Lily (one of the most well-known horticultural crops)	(Li et al., 2013)
Heilongjiang, China	Withered tips, chlorosis, stunting, yellow leaves, leaf drop, root-growth inhibition, and crown rot	Rice (the staple food of over half of the world's population.)	(Li et al., 2019)
Gansu, China	Developed white tufts of mycelium on the radicles of germinating seeds causing them to quickly die	Seeds of Stipa bungeana and Lespedeza davurica	(Chen et al., 2017)
Gansu, China*	Stem and root rot, vessels showed a brown to dark brown discoloration, plus a progressive yellowing and wilting of leaves from the base.	Lanzhou lily (an important bulb edible crop)	(Shang et al., 2014)
Italy	Fusarium Head Blight	Kernels of durum wheat	(Amato et al., 2015)
The salt lake of Xinjiang, China	As endophytic fungi	*Salicornia bigelovii* (a salt-tolerant land plant that	(Zhang et al., 2015)

		grows on salt marshes）	
Argentina	Affect yield and grain quality	Winter cereal crop	(Castañares et al., 2010)
Iowa, US	Fusarium blight of soybeans	Soybean	(Nyvall, 1976)
New Zealand	Infertility in sheep feeding on pasture grass (mycotoxin contamination)	Pasture grass and soil	(Bosch et al., 1989)
Pyeongchang，Korea	Postharvest fruit rot	Pumpkin	(Aktaruzzaman et al., 2018)
The northern Great Plains, US	Root rot	Soybean	(Yan & Nelson, 2020)
Ontario, Canada	Fusarium root rot complex (major soybean disease), root rot severity and reductions in seedling emergence, plant height and root dry weight	Soybean	(Zhang et al., 2010)
Poland	Fusarium head blight	Winter wheat	(Lenc, 2015)
Alberta, Canada	Root rot, which usually results in the occurrence of dead or dying plants in mid-to-late summer and severe reductions in yield	Soybean	(Zhou et al., 2018)
North China	Reduce the quantity of harvested forage	Alfalfa (a popular cultivated crop for forage)	(Cong et al., 2016)
France	Potential risk of mycotoxin contamination in grains	Durum wheat and spring barley	(Orlando et al., 2019)
Vojvodina，Serbia	Infected garlic bulbs occurred in storage and warehouses	Garlic bulbs	(Ignjatov et al., 2016)
Ilam, Iran	As endophytic fungi on Persian oak trees	Trees and seedlings of oak trees	(Alidadi et al., 2019)
Syria	Fusarium head blight	Wheat	(Alkadri et al., 2013a)
Iowa, US	Root rot	Soybean roots	(Arias et al., 2013)
Minnesota, US	Caused low amounts of seed rot	Alfalfa	(Berg et al., 2017)
Gansu, Hebei,	Typical root rot	Sugar beet	(Cao et al., 2018)

Heilongjiang, Inner Mongolia, China			
Taizhou, Lianyungang, China	Fusarium head blight	Rice	(Dong et al., 2020)
Alberta, Saskatchewan, and Manitoba, Canada	Root, crown, and foot rot of field pea	Pea	(Esmaeili Taheri et al., 2017)
The Central Region, France	Root and collar rot in carrot seed parcels	Carrot	(Le Moullec-Rieu et al., 2019)
Hubei, china	Rot of Broccoli heads post-harvest	Broccoli	(Zhao et al., 2017)
Gansu and Ningxia, China*	Severe root rot	Wolfberry	Uwaremwe et al., 2020)

*indicates our research data.
