# Supplementary material for: Distribution, pathogenicity and disease control of Fusarium tricinctum
Source: Front Microbiol. 2022 Jul 26;13:939927. doi: 10.3389/fmicb.2022.939927 (PMC9360978; doi:10.3389/fmicb.2022.939927)

**Fig. S1**. Chemical structures of enniatins and beauvericin: Enniatin A: R 1 = R2 = R3 = sec-butyl; Enniatin Al: R 1 = isopropyl, R2 = R3 = sec-butyl; Enniatin B: R, = R2 = R3 = isopropyl; Enniatin B1: R, = R2 = isopropyl, R3 = sec-butyl; Beauvericin:R, = R, = R3 = benzyl.

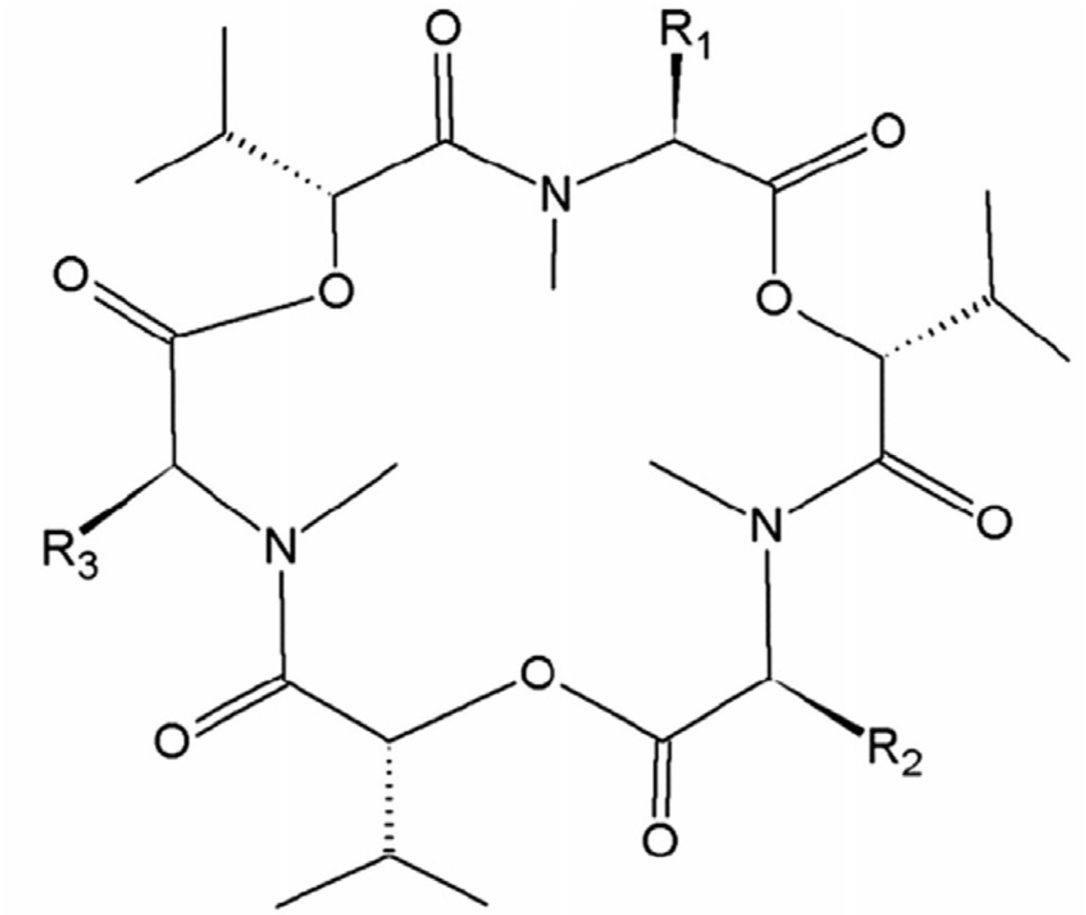

**Fig. S2.** Chemical structure of moniliformin

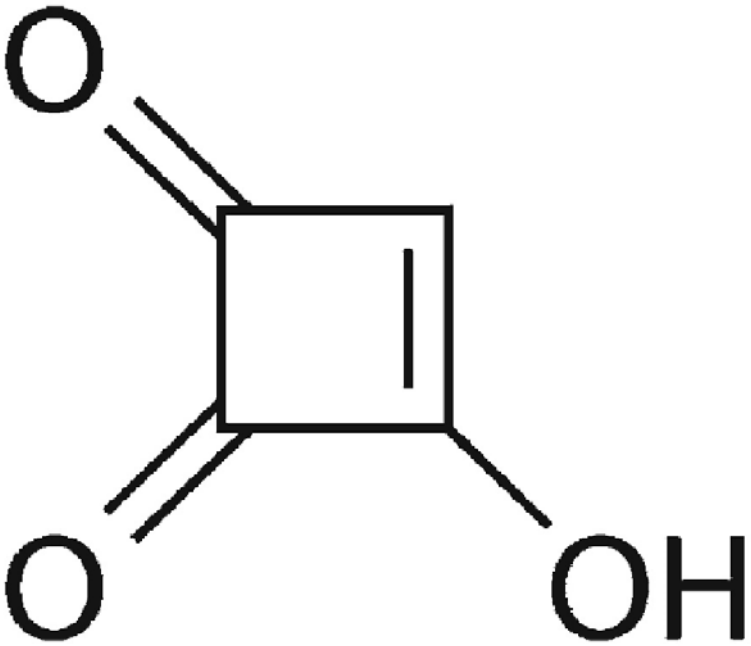

Supplement: Supplementary file 2 [file Data_Sheet_2.PDF]
